# Uncovering trends in training progression for a national cohort of psychiatry trainees: discrete-time survival analysis

**DOI:** 10.1192/bjo.2021.958

**Published:** 2021-06-28

**Authors:** Milou E.W.M. Silkens, Shah-Jalal Sarker, Asta Medisauskaite

**Affiliations:** Research Department of Medical Education, UCL Medical School, University College London, UK; Research Department of Medical Education, UCL Medical School, University College London, UK; and UCL Queen Square Institute of Neurology, School of Life & Medical Sciences, University College London, UK; Research Department of Medical Education, UCL Medical School, University College London, UK

**Keywords:** Education and training, psychiatry training, training progression, discrete survival analysis, UKMED

## Abstract

**Background:**

The global rise in mental health issues calls for a strong psychiatry workforce. Yet, psychiatry training worldwide is facing recruitment challenges, causing unfilled consultant posts and possibly threatening the quality of patient care. An in-depth understanding of trainees’ progression through training is warranted to explore what happens to recruited trainees during training.

**Aims:**

To uncover current trends in psychiatry trainees’ progression through training in the UK.

**Method:**

This national retrospective cohort study with data from the UK Medical Education Database used discrete-time survival analysis to analyse training progression for those trainees who started their core psychiatry post in 2012–2017 (2820 trainees; 59.6% female, 67.6% UK graduates (UKGs)). The impact of sociodemographic characteristics on training progression were also investigated.

**Results:**

The overall probability of completing training in 6 years (minimum years required to complete psychiatry training in the UK) was 17.2% (ranging from 4.8% for non-UKG females to 29% for UKG males). The probability to not progress was highest (57.1%) from core to specialty training. For UKGs, trainees from ethnicities other than White, trainees with a disability, and trainees who had experienced childhood social deprivation (measured as entitlement to free school meals) had a significantly (*P* ≤ 0.02) lower probability of completing training in 6 years.

**Conclusions:**

Less than one in five psychiatry trainees are likely to complete training in 6 years and this probability varies across groups of doctors. Completing psychiatry training in 6 years is, therefore, the exception rather than the norm and this has important implications for trainees, those planning psychiatry workforces or responsible for psychiatry training.

## Background

The global rise in identification of mental health problems^1^ makes accessible and high-quality mental health services increasingly important. As a result, psychiatry is challenged by workforce demands in the UK^2^ and internationally.^3,4^ In 2019, for example, there were 708 (10%) vacant or unfilled psychiatry consultant posts in England, a number that has tripled from 220 in 2013.^2^

After the Centre for Workforce Intelligence identified that psychiatry training programmes were struggling to recruit sufficient numbers of trainees,^5^ the Royal College of .Psychiatrists developed a 5-year strategy in 2012 to increase recruitment of trainees in psychiatry.^6^ The number of doctors working within mental health services in the UK, however, did not change between 2009 and 2018.^7^ Although recent social media campaigns seem promising in improving the number of psychiatry trainees recruited,^8^ this may not result in an increased number of consultants as the pathway to consultancy is long and challenging. Therefore, to work towards insight into how to best increase the number of psychiatrists, an in-depth understanding of trainees’ progression through training is warranted.

## Aims

The insight needed, however, is currently lacking and this study is the first to apply discrete-time survival analysis to a national cohort of psychiatry trainees to identify the proportion of trainees that are progressing to the next training level each year as well as those that do not. In doing so, the proportion of trainees that complete psychiatry training in 6 years (i.e. the minimal number of years required to complete training in the UK) and the stages of training at which trainees are most likely to not progress will be revealed.

Trainees’ progression is expected to be dependent on demographic and socioeconomic variation and therefore this study sets out to also investigate the impact of such variation on trainees’ progression through training. By providing an in-depth exploration of trainees’ progression and the factors that have an impact on this progression our study may ultimately contribute to achieving a better understanding of successful completion of training programmes in psychiatry and other specialties facing similar challenges such as general practice.^9^

## Method

### Study setting

This study was designed as a national retrospective cohort study within the setting of psychiatry training in the UK. Psychiatry training takes a minimum of 6 years to complete: trainees complete 3 years of core psychiatry training (CT1–CT3) and subsequently apply in open competition to do 3 years of specialty psychiatry training (ST4–ST6). Some specialty training programmes are an exception to this and can take more than 3 years.

In this study we defined CT1 trainees who reached ST6 training level in the minimum required training time as trainees who complete training within 6 years’ time.

### Ethics

All procedures contributing to this work comply with the ethical standards of the relevant national and institutional committees on human experimentation and with the Helsinki Declaration of 1975, as revised in 2008, and were approved by the Queen Mary's Ethics of Research Committee (University of London) on the 16 January 2017.^10^

### Study population and design

Data were provided by the UK Medical Education Database (UKMED) Development Group (permission granted in February 2019) and accessed by the researchers through the Safe Haven of the Health Informatics Centre at Dundee University. The UKMED is a large national data-set that provides routinely collected data on the undergraduate and postgraduate performance of medical students and trainees in the UK.^11^ For the present study we used UKMED data that were available from 2012 till 2018. We analysed the progression of those psychiatry trainees who were in a CT1 post between 2012 and 2017. We also explored a subcohort of trainees in CT1 in 2012 or 2013, which allowed full follow-up until ST6 (no right censoring) as sensitivity analyses and two UKG subcohorts (those in a CT1 post between 2012–2017 and 2012–2013) as most relevant demographic data were available for UKGs only.

### Variables

We used National Training Survey (NTS) data within the UKMED to investigate trainees’ progression through training. The NTS data contains self-reported trainee-level data that were collected annually around March to May. The variable ‘year’ in this paper represents the year during which data were collected (for example 2012 represents academic year 2011/2012).

A selection of demographic variables collected in the UKMED were used to examine their impact on trainees’ progression. Participants’ gender (female or male) and the region of their Primary Medical Qualification (PMQ: UK/non-UK) was used for analysis with the main cohort and all subcohorts. For UKGs specifically we analysed the following variables (variables that overlapped or with more than 25% missing values were excluded):

- trainees’ ethnicity (White/Black and Minority Ethnic (BME)),
- type of school (private/state),
- status at the time of entry to medical school (graduate/non-graduate),
- disability (yes/no; answer ‘yes’ represents ‘yes’ on the disability variable from the Higher Education Statistics Agency, HESA, and/or limited activities variable from NTS),
- indicators of social background: entitlement to free school meals (social deprivation in childhood, yes/no), parents’ university degree (yes/no; variable was constructed by replacing any missing values in the self-reported variable on parents’ degree from the NTS with data on parents’ degree from the HESA), Index of Multiple Deprivation) which is a neighbourhood-based measure of social deprivation (most deprived/other; the most deprived level combined levels 1 and 2).

More details about variables can be found in the UKMED data dictionary.^12^

### Data preparation

We selected all trainees who started their CT1 post between 2012 and 2017 and investigated whether in the next year they progressed to the next training level (CT2). We then followed-up those trainees who successfully progressed to CT2 in a similar way and so on. From this approach, it follows that we excluded those trainees not progressing to the next training level in the next year from further follow-up. We treated all trainees taking longer than 6 years to complete training in the same way, meaning that this group also included trainees working less than full time (LTFT) that progressed through training slower.

Trainees not progressing may stay in their current training level for subsequent years. It was crucial to identify these trainees as they could potentially bias the cohort. A concrete example of such bias is when a trainee in CT1 in 2012 does not progress to CT2 and stays in CT1 in 2013, which would mean this trainee is duplicated in the data-set by having a record for both years, 2012 and 2013. For each year of our study time frame we identified an average of 54 such trainees and accounted for these cases by including only trainees’ first attempt to progress and removing duplicated data in later years. As a result of the given time frame, it was impossible to account for the training history of trainees who were indicated to be in CT1 in 2012 and therefore a small number of trainees in our sample may have started their CT1 before 2012.

### Statistical analysis

Survival analysis is a widely used method and originally developed to investigate mortality rates in clinical trials. Survival analysis is not widely used in medical education research (see Tiffin et al^13^ for a rare example), but adds much needed rigour to the investigations of medical trainees’ progression. This is because survival analysis is equipped to analyse those cases that are missing data because of the limited study time frame (right censoring) whereas traditional regression underestimates the true time-to-event in the presence of censoring.

We analysed progression data using discrete-time survival analysis (rather than the popular Cox proportional hazards model) that is generally recommended for the estimation of a target event in educational research, especially when the used time metric (i.e. time interval) is large (for example a year or quarter). As most educational phenomena are measured at discrete-time intervals, the use of continuous survival analysis can lead to biased parameters.^14–16^ In a recent study Kim et al^17^ recommended discrete-time models when cases had a smaller number of time points available, larger time intervals were used, for studies with larger sample sizes and for studies with smaller proportions of censored observations. As our study identified events annually using only five time points and used a large sample size, discrete-time survival analysis was the justified way of dealing with tied observations of events. Although in the analysis of the main cohort and the UKG 2012–2017 cohort censoring was not low, the fixed right censoring did not reduce the chance of experiencing many events of non-progression at the same time. Moreover, our additional analysis based on 2012 and 2013 CT1 trainees did not have any right censoring induced by the study design.

We defined ‘survival’ as trainees that completed training in 6 years up to the end of the study time frame (2018). Participants that did not progress to the next training level in the minimum required time at any given point within the time frame of the study were defined as ‘cases’ with an ‘event’. The first time point in the analysis was trainees’ transition from CT1 to CT2 and we examined a total of five time points during which an event could occur (time 1: CT1 to CT2; time 2: CT2 to CT3; time 3 CT3 to ST4; time 4: ST4 to ST5; time 5: ST5 to ST6), thereby identifying any occurring events annually (Table 1). Trainees with fixed right censoring were accounted for until the time of censoring in 2018.^18^

**Table 1:** Number of trainees progressing through training and censored data

CT1 year	Transition					
	CT1 (*n*)	CT2 (*n*)	CT3 (*n*)	ST4 (*n*)	ST5 (*n*)	ST6 (*n*)
2012	525	410	305	130	110	70
2013	465	370	280	115	90	75
2014	470	375	300	140	125	(125)^a^
2015	460	385	335	135	(135)^a^	(135)^a^
2016	430	355	315	(315)^a^	(315)^a^	(315)^a^
2017	465	390	(390)^a^	(390)^a^	(390)^a^	(390)^a^

CT, core psychiatry training; ST, specialty psychiatry training.

a. Represent data outside of the study time frame that were censored (*n* = number censored).

All statistical analyses were performed in Statistical Package for the Social Sciences (SPSS) v24 and Microsoft Excel. We constructed a person–period data-set^19^ in which each participant had a row in the data-set corresponding to each year they were at risk of experiencing the event (not progressing). A dummy variable was created for each of the five time points and the set of dummies was entered as intercepts in a univariate binary logistic regression predicting trainees’ progression. We calculated and plotted baseline hazards and survival curves for the main cohort and the subcohorts manually in Microsoft Excel.^20^

For the main cohort and the 2012–2013 subcohort multivariable analysis was performed in SPSS using only the variables gender and region of PMQ and corresponding hazard probabilities, and survival curves were calculated and plotted. For the UKG subcohorts we added available socioeconomic variables (see section ‘Variables’) to the multivariable analysis and presented odds ratios (ORs), the 95% confidence interval and *P*-values to indicate the impact of the covariates on trainees’ progression. As covariates were not time-dependent, the hazard ratios of covariates were the same for each time interval and the proportionality assumption was met.^21^ An interaction term between covariates was added to the final model when the interaction term was significant or whether the −2 Log Likelihood (−2LL) improved substantially (Δ−2LL = 4).

We also calculated the more frequently used Life tables and Kaplan–Meier survival curves as a sensitivity analysis to see whether these methods would be comparable with our more appropriate discrete-time approach. The results of this analysis are provided in the Supplementary File 1 (supplementary Table 1 and supplementary Figs 1 and 2) available at https://doi.org/10.1192/bjo.2021.958.

### Data presentation

The following statistical disclosure controls were applied as HESA data were used (source: https://www.hesa.ac.uk/about/regulation/data-protection/rounding-and-suppression-anonymise-statistics): 0, 1, 2 were rounded to 0 and all other numbers were rounded to the nearest multiple of 5. Percentages based on fewer than 22.5 individuals were suppressed, and averages based on seven or fewer individuals were suppressed.

## Results

### Study population

The main cohort contained 2820 trainees of whom 1680 (59.6%) were women and 1905 (67.6%) UKGs (Supplementary File 2, Supplementary Table 2). Similar percentages of women (615; 62.2%) and UKGs (625; 63.1%) were observed in the 2012–2013 subcohort of 990 trainees. Distributions of demographic characteristics in the subcohorts of UKGs and non-UKGs are presented in Supplementary Table 2.

### Baseline hazard and survival probabilities

**Fig. 1:**
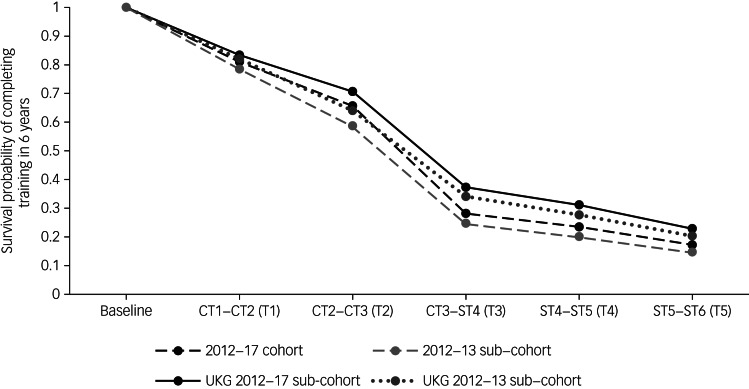
Survival curves for the main 2012–2017 cohort, the 2012–2013 subcohort and the UK graduate (UKG) subcohorts.

Table 2 provides the unstandardised coefficients from the discrete-time survival analysis and the derived hazards (representing no progression to the next training level) and survival probabilities (representing progression to the next training level), which are plotted in Fig. 1. A negative coefficient means that more trainees progress than not whereas a positive coefficient means that more trainees do not progress.

**Table 2:** Unstandardised coefficients, baseline hazards (probability of not progressing) and cumulative survival probabilities (probability of progressing to the next training level) for the main cohort and the two subcohorts based on discrete-time survival models

	Unstandardised coefficient	Baseline hazards	Cumulative survival
2012–2017 cohort			
CT1–CT2	−1.451	0.190	0.810
CT2–CT3	−1.452	0.190	0.656
CT3–ST4	0.287	0.571	0.281
ST4–ST5	−1.616	0.166	0.235
ST5–ST6	−1.013	0.266	0.172
2012–2013 subcohort			
CT1–CT2	−1.295	0.215	0.785
CT2–CT3	−1.088	0.252	0.587
CT3–ST4	0.319	0.579	0.247
ST4–ST5	−1.465	0.188	0.201
ST5–ST6	−1.013	0.266	0.147
UKG 2012–2017 subcohort			
CT1–CT2	−1.613	0.166	0.834
CT2–CT3	−1.712	0.153	0.706
CT3–ST4	−0.113	0.472	0.373
ST4–ST5	−1.617	0.166	0.311
ST5–ST6	−1.016	0.266	0.229
UKG 2012–2013 subcohort			
CT1–CT2	−1.522	0.179	0.821
CT2–CT3	−1.264	0.220	0.640
CT3–ST4	−0.130	0.468	0.341
ST4–ST5	−1.464	0.188	0.277
ST5–ST6	−1.016	0.266	0.203

UKG, UK graduate.

The coefficients for the 2012–2017 cohort showed that for each transition in training more people progress than not, except for the transition from core to specialty training where more trainees did not progress to the next training level. The baseline hazards for the 2012–2017 cohort indicated that the probability to not progress was highest for the transition from CT3 to ST4 (57.1%). The cumulative survival probability for this cohort showed that the probability of completing training in 6 years was 17.2%.

The results for the 2012–2013 subcohort were very similar, showing a comparable pattern in the survival and hazard probabilities, and similarly showing the highest hazard probability at the transition from CT3 to ST4 (hazard  0.579). The cumulative survival probability for the 2012–2013 subcohort showed that the probability of completing training in 6 years was 14.7%.

The coefficients for the UKG 2012–2017 and UKG 2012–2013 subcohorts were all negative, indicating that for each transition in training more people were progressing than not. For both subcohorts the baseline hazard was highest for the transition from CT3 to ST4. The baseline survival probability for the UKG 2012–2017 subcohort showed that 22.9% of the trainees were expected to complete training in 6 years and for the UKG 2012–2013 subcohort this was 20.3%.

### Multivariable analysis for the main cohort and the 2012–2013 subcohort

The odds to complete training in 6 years significantly differed between men and women as well as between UK and non-UK graduates in the 2012–2017 cohort (χ^2^(7) = 2390.004, *P* < 0.001; *R*^2^ = 0.307; Supplementary Table 3). The odds ratio for women was OR = 0.671 (95% CI 0.593–0.759) and for non-UKGs was OR = 0.463 (95% CI 0.408–0.525). Therefore, the odds for completing training in 6 years were respectively 1.49 (1/0.671) times and 2.16 (1/0.463) times higher for men and UKGs trainees than their respective counterparts. Supplementary Table 3 shows similar findings for the 2012–2013 subcohort.

**Fig. 2:**
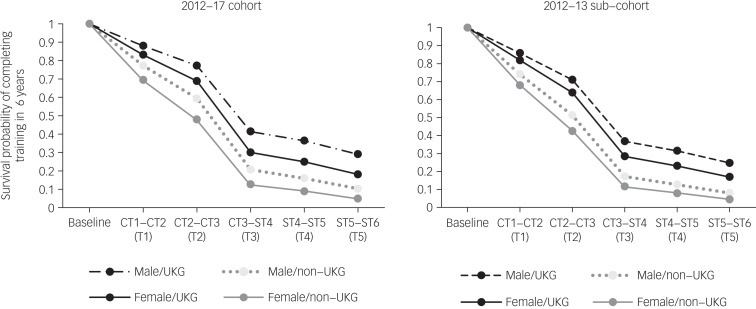
Survival curves for the multivariable analysis for the 2012–2017 cohort and the 2012–2013 subcohort. UKG, UK graduate.

The interaction term between gender and PMQ was not significant (*P* = 0.850) and did not improve the −2LL and was therefore not included in the model. The differences in the survival curves between UKGs/non-UKGs and men/women are visualised in Fig. 2. The lines (for categories of factors) do not cross, which supports our expectation of no significant interaction effect. The hazards indicated that the probability to not progress is highest for non-UKGs and specifically for female non-UKGs (Supplementary Table 4). The probability of completing training in 6 years was 4.8% for non-UKG women, 10.3% for non-UKG men, 18.1% for UKG women and 29% for UKG men. Similar results were found for the 2012–2013 subcohort (Supplementary Table 4). The probability of completing training in 6 years varied from 4.4% for non-UKG women to 24.7% for UKG men.

Sensitivity analysis based on Life-tables and Kaplan–Meier analysis showed similar results as the discrete-time survival analysis and are presented in Supplementary File 1 (supplementary Table 1 and supplementary Figs 1 and 2).

### Multivariable analysis for the UKG subcohort

For the UKG 2012–2017 subcohort gender, ethnicity, disability, and entitlement for free school meals were independently associated with the probability to complete training in 6 years (Table 3). The odds of completing training in 6 years was lower for female trainees (OR = 0.725, 95% CI 0.604–0.871), but 1.295 times higher for White trainees, 2.766 greater for trainees without a disability and 1.936 greater for trainees who were not entitled to free school meals during childhood.

**Table 3:** Multivariable discrete-time survival (probability of completing training in 6 years) analysis results for the UK graduate (UKG) 2012–2017 cohort and the UKG 2012–2013 subcohort

Predictor (reference category)	UKG 2012–2017 subcohort^a^ (*n*^b^ = 1406)						UKG 2012–2013 subcohort^a^ (*n*^b^ = 466)					
	OR	95% CI	*P*	χ^2^(13) (*P*)	*R*^2^ (Cox & Snell)	*R*^2^ (Nagelkerke)	OR	95% CI	*P*	χ^2^(13) (*P*)	*R*^2^ (Cox & Snell)	*R*^2^ (Nagelkerke)
Gender (female)	**0**.**725**	**0.604–0.871**	**0**.**001**				0.918	0.707–1.192	0.521			
Ethnicity (White)	**1**.**295**	**1.053–1.593**	**0**.**014**				1.108	0.817–1.503	0.510			
Index of Multiple Deprivation (most deprived)	0.856	0.685–1.070	0.172				**0**.**676**	**0.486–0.940**	**0**.**020**			
Graduate on entry (graduate)	0.819	0.653–1.027	0.840				0.766	0.543–1.082	0.130			
Disability (no)	**2**.**766**	**1.968–3.888**	**< 0.001**				**2**.**085**	**1.308–3.332**	**0**.**002**			
Free school meals (no)	**1**.**936**	**1.439–2.605**	**< 0.001**				1.588	0.976–2.582	0.063			
Type of school (state)	0.935	0.758–1.154	0.534				0.952	0.706–1.284	0.748			
Parent degree (yes)	1.010	0.832–1.226	0.923				1.020	0.764–1.363	0.892			
Model statistics				604.613 (<0.001)	0.373	0.497				577.607 (<0.001)	0.316	0.421

Results in bold are significant.

a. Dummy variables representing time variables were entered into the model as intercepts.

b. The *n* is smaller for the multivariable analysis because of exclusion of participants with missing variables for any of the covariates.

The UKG 2012–2013 subcohort, however, showed no differences in gender, ethnicity and free school meals. Results did confirm that the probability of completing training in 6 years was higher for trainees without a disability (OR = 2.085; 95% CI 1.308–3.332) and showed that trainees from more deprived areas were at a higher odd of not progressing (OR = 0.676; 95% CI 0.486–0.940). No indications for multicollinearity were found that could explain the differences between cohorts, yet the variables ‘free school meals’ and ‘Index of Multiple Deprivation’ may have an impact on each other as both are indicators of trainees’ socioeconomic status.

## Discussion

To explore trends in psychiatry trainees’ progression through training, this study (a) investigated the probability of trainees progressing at each stage of their training as well as completing training in the minimum required time of 6 years, (b) identified the stages of training at which trainees are most likely to not progress at once, and (c) assessed the impact of various sociodemographic characteristics on trainees’ progression. Using discrete-time survival analysis as a methodological approach unique to the field of medical education, this study investigated a large national retrospective cohort to uncover novel insights in psychiatry trainees’ career progression.

### General trends in psychiatry trainees’ career progression

Our study revealed that trainees’ probability to progress to the next level of their training varied per training level. For all our cohorts, trainees were least likely to progress at the transition from core to specialty training (for example 57% of trainees in the main cohort did not progress from CT3 to ST4). This is in line with previous findings in a publication from the Centre for Workforce Intelligence,^5^ speculating that the trend may be attributable to low pass rates of the three examinations that trainees are required to pass during core training in order to progress to specialty training. Furthermore, some trainees may fail to secure a training post at ST4, even after successfully completing CT3 as the availability of ST4 posts may vary per region.

More novel, however, are our findings showing that there is a substantial proportion of trainees who do not progress during other stages of training (for example approximately one-fifth of trainees do not progress from CT1 to CT2). Furthermore, only a small proportion of psychiatry trainees are expected to complete training in 6 years (17.2% in the main cohort), meaning that most trainees that start their training either drop-out or take longer than 6 years to complete their training. This may be because of, as mentioned above, low pass rates for exams, but also trainees choosing to work LTFT or taking breaks to accommodate, for example, maternity/paternity leave, personal or professional development needs, or to cope with psychological pressures (for example arising from experienced stigma,^22^ inflexibility of training^23^ or stress^24^). Taking longer than 6 years to complete training can, therefore, be a well-considered and planned decision (for example working LTFT or taking career breaks) as well as unplanned (for example failing exams). Alternatively, trainees may also choose to leave psychiatry training and switch, for example, to another specialty.

Despite the fact that our study shows that just under 100 trainees are expected to complete training in 6 years each year, annual numbers provided by the Royal College of Psychiatrists show that approximately 600 trainees reach ST6 level each year.^2^ This means that the majority of trainees completing training each year are trainees that took longer than 6 years to reach ST6.

### Group-specific trends: the role of sociodemographics

In addition to our findings on general trends in trainees’ career progression, the analysis of sociodemographic characteristics of trainees contributes to a better understanding of progression through training by revealing group-specific trends. The current study found that the odds of completing training in 6 years were significantly lower for women and individuals who were non-UKGs. Combining gender and PMQ, we found that male UKGs had the highest probability of completing training in 6 years (29%) and female non-UKGs the lowest (4.8%), implying that non-UKG trainees were less likely to complete their training in 6 years especially when they were women.

Within the UKG 2012/2017 subcohort, women trainees also had a significantly lower odds of completing training in 6 years (OR = 0.725) than men trainees, whereas the odds of completing training in 6 years were greater for White trainees (1.295 times), trainees without disability (2.766 times) and trainees who had not had entitlement to free school meals during childhood (1.936 times). Although the impact of gender, ethnicity and free school meals were not significant in the UKG 2012/2013 subcohort and the Index of Multiple Deprivation did become significant, odds ratios were comparable in both cohorts. Variations in significance may come from differences in sample size and minor variations in distributions of predictors because of missing values.

From this it follows that some groups of doctors are less likely to complete training in the minimum time required (6 years), which may be indicative of differential attainment that occurs for different reasons among different groups.

For the differences found between UKGs and non-UKGs and in ethnicity there is convincing evidence suggesting that UKGs and doctors of White ethnicity perform better in postgraduate examinations,^25–28^ which may explain a higher odds of progression in 6 years for these groups of trainees. For psychiatry training specifically, differences in the region of PMQ and ethnicity were identified in performance for the clinical examination.^29^ Research across a range of specialties also reveals various challenges that non-UKGs have to deal with in their career paths, such as cultural differences,^30^ a lack of trust from supervisors, and separation from family support, which makes them more vulnerable to stress and burnout^28^ and, in turn, may increase their odds to drop-out of or take longer to progress during psychiatry training. Similarly to non-UKGs group, BME trainees might face more challenges during their training compared with White trainees.^28^

When explaining gender differences, family responsibilities (such as more often living with people over 60 years^31^) and work challenges (such as intimidation, harassment and discrimination^32^) might cause women trainees to be at higher odds of leaving training, taking breaks or choosing flexible training pathways to deal with those challenges. Indeed, part-time working is more common for women (42%), whereas 79% of male doctors work or train full time;^33^ and women are slightly more likely to change their career intentions (28% *v*. 24%).^33^ Although evidence on the particular challenges for low socioeconomic status trainees is less substantial, there are indications that unique challenges for this group of trainees also exist (such as related to costs, perception that a medical career is for ‘posh people’^34^) that might make it more difficult for them to progress through training in 6 years.

Finally, in the report ‘Disability equality in the medical profession’ the British Medical Association^35^ revealed that when disabilities are viewed in a traditional sense, a disability is considered to be a physical or mental impairment, or something that doctors try to heal^35^ as opposed to something that doctors themselves can have. Such a perception might challenge trainees with disabilities careers and may link to the barriers doctors with disabilities are reporting: poor support,^36^ inflexible working patterns, unsympathetic colleagues and stigma.^35^ As a result of these challenges trainees might take breaks or change their careers, which Smith et al^36^ reports as being planned ahead or imposed.

The medical workforce, however, is changing rapidly. The number of international medical graduates working as psychiatry consultants, for example, increased by 140% from 1998 to 2012,^5^ in 2019 44% of psychiatry consultants were women,^2^ and the number of applicants with disabilities applying to medical schools doubled from 268 in 2001 to 598 in 2005.^35^ This changing workforce may conflict with the traditional views on diversity in the medical workforce, meaning that the training model that was created for ‘a traditional doctor’ is not effective anymore. This may mean that, in order to retain psychiatry trainees, the way in which psychiatrists are trained may need to change; as well as the support provided during their careers.

### Strengths and limitations

The study draws from the national UKMED, which is the most complete overview of trainees’ progression available in the UK. This allowed for elaborate additional analysis using the 2012–2013 cohorts without censored data, which is a unique strength of this study as for those cohorts we were certain how many trainees completed training in 6 years. We were also able to confirm our results using more traditional continuous time survival analysis techniques as sensitivity analysis for the more appropriate, but also more novel, discrete-time survival analysis. This supported the concurrent validity of our results.

Nevertheless, as UKMED data were only available from 2012 onwards the current study was not able to take into account the training history of trainees starting their CT1 in 2012. This means that, on average, there will be 54 trainees in our data-set included as first-time trainees, whereas in reality they started their CT1 before 2012 (see section ‘Data preparation and statistical analysis’) and hence are left censored. We estimate that the impact of this left censoring in our data can be considered negligible considering the sample size. However, any impact of this limitation on study outcomes would mean that we slightly overestimate trainees’ probability to complete training in 6 years.

Another limitation is that only a selection of sociodemographic variables were used in the current study, whereas the UKMED provides numerous alternatives or possible additions that have the potential to further unravel underlying reasons for trainees’ various pathways through training (for example working LTFT, age and deaneries). However, because of factors such as >25% of missing data and/or inconsistencies in the data-set over time these variables pose substantial challenges for research and were therefore excluded from the current study.

Furthermore, approximately 25% of available ‘cases’ in both UKG cohorts were left out from our analyses as they had missing data on one or more of the sociodemographic variables selected for our analyses. Although after deletion of these cases the remaining sample sizes were still sufficiently large, there may have been an impact on results (for example significant results are harder to replicate in smaller samples, which may be an explanation for the variations in results).

### Implications for research and practice

This study gives two core leads for future research. First, to explore what proportion of trainees not completing training in 6 years still eventually complete training; this would help to get a more complete picture of trainees’ progression through training. This would be particularly useful as trainees who work LTFT might be progressing successfully through training, but just at a slower pace because of their working patterns.

Second, although the current study provides evidence that several groups of trainees have a higher likelihood to complete training in 6 years than other groups, the question why still remains. We call for research into a more in-depth exploration of underlying reasons for trainees’ various progression patterns. Research could focus on, for example, the occurrence of life events (such as caring responsibilities or parenthood), detailed demographic differences (for example age-related differences) and psychiatry training specific reasons (for example high demand on trainees’ well-being because of the patient population). We also recommend looking at core training and specialty training separately as we expect underlying reasons for progression to vary per training period.

The study also provides two crucial recommendations for practice. First, from the main finding that most trainees do not complete their training in 6 years, it follows that it is unrealistic to expect that an increase in the recruited number of trainees will result in an increase in doctors working in consultant posts after 6 years. This should be considered when planning the workforce in psychiatry. It also has a significant impact on trainees’ expectations when starting their training, for example regarding the time of completion as well as the flexibility of their training. Second, the findings that some groups of trainees have a lower probability to get through training in 6 years than others has significant equality, diversity and inclusion implications. In line with what was discussed in the section above, stakeholders responsible for psychiatry training should consider these new findings when planning training provision.

## Data Availability

The data that support the findings of this study are not publicly available due to containing information that could compromise the privacy of participants in the research. Data are managed by the UKMED group.

## Supplementary material

For supplementary material accompanying this paper visit http://dx.doi.org/10.1192/bjo.2021.958. click here to view supplementary material
