# Supplementary material for: Uncovering trends in training progression for a national cohort of psychiatry trainees: discrete-time survival analysis
Source: BJPsych Open. 2021 Jun 28;7(4):e120. doi: 10.1192/bjo.2021.958 (PMC8269924; doi:10.1192/bjo.2021.958)
Supplement: Supplementary file 1 [file S2056472421009583sup001.docx]

# Supplementary material

## Supplement File 1: Survival analysis for the main cohort and 2012/2013 sub-cohort using life tables and Kaplan-Meier curves

Life-tables were used to estimate the probability of an event for trainees at risk at any of the five time points. Kaplan–Meier survival curves were used to assess the impact of gender and region of PMQ on trainees’ progression. We used the log-rank test with Breslow’s correction for ties.

The life table is presented in Supplementary Table 1. From the 2819 psychiatry trainees who started their training between 2012 and 2017 just 269 reached ST6 in six years (20%). The probability to not progress was the highest for the transition from core to specialty training (68%). Similar results were found for uncensored cohort of trainees starting their training in 2012-13. From the 991 psychiatry trainees who started CT1 just 146 reached ST6 in six years (12%) with the probability of 81% to not progress from core to specialty training.

**Supplementary Table 1** Life table based on 2012-17 and 2012-13 cohorts

|  | **Number of** | | | **Proportion of** | | |
| --- | --- | --- | --- | --- | --- | --- |
|  | **Trainees progressing** | **Trainees non-progressing** | **Censored data** | **Survival rates (probability of trainees progressing each year)** | **Cumulative survival rates (probability of completing training six years)** | **Hazard rates (probability of not progressing)** |
| **2012-17 cohort** | | |  |  |  |  |
| CT1 | 2819 | na | na | na | 1 | na |
| CT1-CT2 | 2284 | 535 | 0 | 0.81 | 0.81 | 0.21 |
| CT2-CT3 | 1533 | 359 | 392 | 0.83 | 0.67 | 0.19 |
| CT3-ST4 | 523 | 697 | 313 | 0.49 | 0.33 | 0.68 |
| ST4-ST5 | 322 | 64 | 137 | 0.86 | 0.28 | 0.15 |
| ST5-ST6 | 269 | 53 | 269 | 0.72 | 0.20 | 0 |
| **2012-13 sub-cohort** | | |  |  |  |  |
| CT1 | 991 | na | na | na | na | na |
| CT1-CT2 | 778 | 213 | na | 0.79 | 0.79 | 0.24 |
| CT2-CT3 | 582 | 196 | na | 0.75 | 0.59 | 0.29 |
| CT3-ST4 | 245 | 337 | na | 0.42 | 0.25 | 0.81 |
| ST4-ST5 | 199 | 46 | na | 0.81 | 0.20 | 0.21 |
| ST5-ST6 | 146 | 53 | na | 0.58 | 0.12 | 0 |

The Kaplan-Meier survival curves of overall data for two cohorts is presented in Supplementary Figure 1. Trainees are progressing without delays on average for 3 (CI 95%: 2.937-3.063) years (2012-13 sub-cohort: 3 CI 95%: 2.921-3.079).


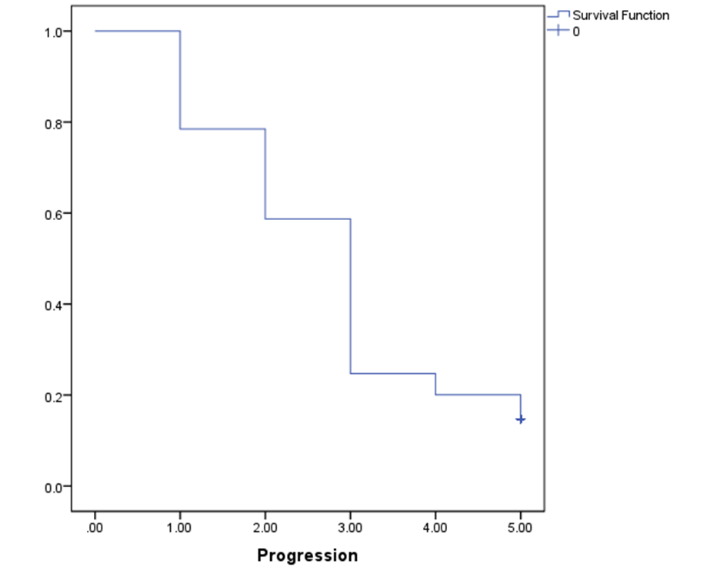

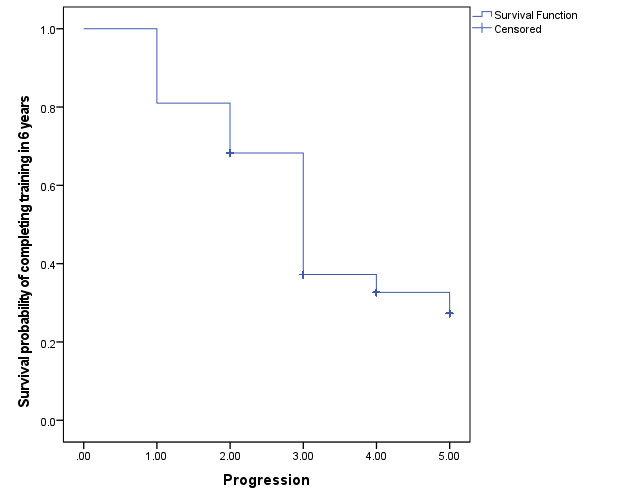


a) b)

**Supplementary Figure 1.** Progression analysis using Kaplan-Meier survival curves: a) overall 2012-17 data; b) overall 2012-13 sub-cohort data.

The 2012-17 cohort analysis showed that male trainees were progressing longer till the event (none-progression to the next training level) than female (log rank test: X^2^(1)=38.567, *p*<.001) and UKGs were progressing longer than non-UK graduates (log rank test: X^2^(1)=149.877, *p*<.001). The probability of completing training in six years was 7% for non-UKG females, 11% for non-UKG males, 20% for UKG females and 36% for UKG males (log rank test: X^2^(3)=190.35, *p*<.001; Figure 2.a).


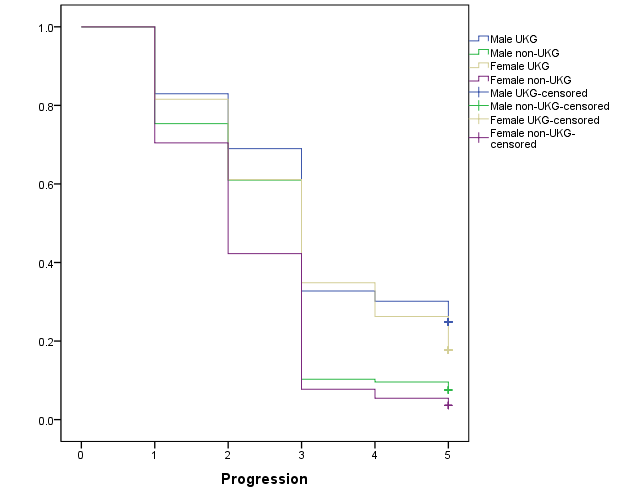

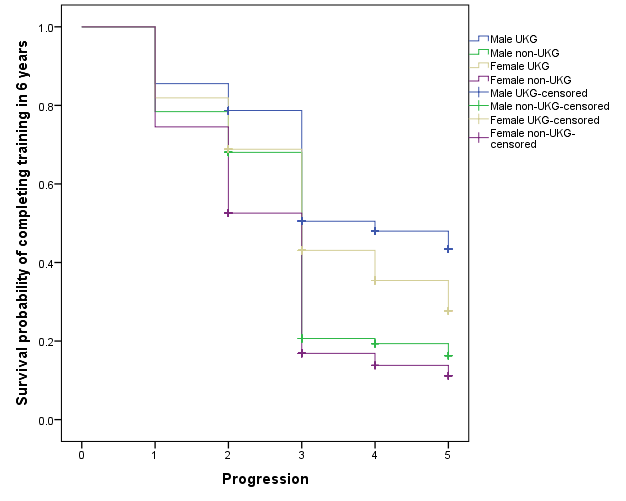
The analysis of 2012-13 sub-cohort without censoring confirmed these findings showing differences in progression between male and female trainees (log rank test: X^2^(1)=7.662, *p*=.006); UKGs and non-UKGs (log rank test: X^2^(1)=62.331, *p*<.001). The probability of completing training in six years was 3% for non-UKG females, 6% for non-UKG males, 13% for UKG females and 21% for UKG males (X^2^(3)=74.212, *p*<.001; supplementary Figure 2.b).

1. b)

**Supplementary Figure 2.** Progression analysis using Kaplan-Meier survival curves: a) according to gender and PMQ in 2012-17 cohort; b) according to gender and PMQ in 2012-13 sub-cohort.

In summary, Life-tables and Kaplan-Meier analysis showed similar results as the discrete-time survival analysis. The cumulative survival probability of completing training in six years for the main cohort was 20.0% (corresponding discrete-time estimate: 17.2%) and for the 2012-13 sub-cohort 12% (corresponding discrete-time estimate: 14.7%). The difference in estimates is likely due to the violation of the assumption of continuous survival analysis that each event occurs at separate point of time (no ties). This justifies the appropriateness of discrete-time survival analysis because of the concentration of ties events in five time points. The sensitivity analysis also confirmed that the probability to complete training in six years was the lowest for female non-UKGs and the highest for male UKGs.

## Supplementary File 2: Tables

**Supplementary Table 2** Detailed study population characteristics for the main cohort and 2012-13, and the UKG/non-UKGs sub-cohorts.

| **Study characteristics** | **2012-17 cohort (N=2820)** | **2012-13 sub-cohort (N=990)** | **UKG 2012-17 sub-cohort (N=1905)** | **UKG 2012-13 sub-cohort (N=625)** | **Non-UKG 2012-17 sub-cohort (N=910)** | **Non-UKG 2012-13 sub-cohort (N=365)** |
| --- | --- | --- | --- | --- | --- | --- |
| Data points in person-period dataset*, N* | 6515 | 2795 | 4600 | 1924 | 1915 | 815 |
| Progression at the end of study timeframe |  |  |  |  |  |  |
| Progression in six years, *N (%)* | 145 (5.2%) | 145 (14.7%) | 125 (6.7%) | 125 (20.3%) | 20 (2.1%) | 20 (5.2%) |
| Events during study period, *N (%)* | 1710 (60.6%) | 845 (85.3%) | 1030 (53.9%) | 500 (79.7%) | 680 (74.5%) | 345 (94.8%) |
| Censored, *N (%)* | 965 (34.2%) | 0 | 750 (39.4%) | 0 | 215 (23.5%) | 0 |
| Gender |  |  |  |  |  |  |
| Female, *N (%)* | 1680 (59.6%) | 615 (62.2%) | 1135 (59.4%) | 395 (63.4%) | 545 (59.9%) | 220 (60.1%) |
| Male *N (%)* | 1140 (40.4%) | 375 (37.8%) | 775 (40.6%) | 230 (36.6%) | 365 (40.1%) | 145 (39.9%) |
| Region of PMQ |  |  |  |  |  |  |
| UKG, *N (%)* | 1905 (67.6%) | 625 (63.1%) | 100% | 100% | 0% | 0% |
| Non-UKG, *N (%)* | 910 (32.4%) | 365 (36.9%) | 0% | 0% | 100% | 100% |
| Ethnicity |  |  |  |  |  |  |
| White, *N (%)* | NA | NA | 1355 (71.1%) | 440 (70.6%) | NA | NA |
| BAME, *N (%)* | NA | NA | 505 (26.5%) | 155 (25%) | NA | NA |
| Missing, *N (%)* | NA | NA | 45 (2.5%) | 30 (4.5%) | NA | NA |
| Type of school |  |  |  |  |  |  |
| Private, *N (%)* | NA | NA | 460 (24.1%) | 150 (23.7%) | NA | NA |
| State, *N (%)* | NA | NA | 1300 (68.2%) | 425 (67.8%) | NA | NA |
| Missing, *N (%)* | NA | NA | 145 (7.7%) | 55 (8.5%) | NA | NA |
| Free school meals |  |  |  |  |  |  |
| Yes, *N (%)* | NA | NA | 140 (7.2%) | 40 (6.4%) | NA | NA |
| No, *N (%)* | NA | NA | 1325 (69.4%) | 445 (71.2%) | NA | NA |
| Missing, *N (%)* | NA | NA | 445 (23.4%) | 140 (22.4%) | NA | NA |
| Parent degree |  |  |  |  |  |  |
| Yes, *N (%)* | NA | NA | 1035 (54.4%) | 340 (54.4%) | NA | NA |
| No, *N (%)* | NA | NA | 510 (26.7%) | 160 (25.9%) | NA | NA |
| Missing, *N (%)* | NA | NA | 360 (18.9%) | 125 (19.7%) | NA | NA |
| Index of Multiple Deprivation (IMD) |  |  |  |  |  |  |
| Deprived (1/2), *N (%)* | NA | NA | 385 (20.2%) | 120 (19%) | NA | NA |
| Other, *N (%)* | NA | NA | 1365 (71.5%) | 445 (71.4%) | NA | NA |
| Missing, *N (%)* | NA | NA | 160 (8.3%) | 60 (9.6%) | NA | NA |
| Graduate on entry |  |  |  |  |  |  |
| Graduate on entry, *N (%)* | NA | NA | 395 (20.7%) | 120 (19%) | NA | NA |
| Non-Graduate on entry, *N (%)* | NA | NA | 1465 (76.8%) | 445 (76.3%) | NA | NA |
| Missing, *N (%)* | NA | NA | 50 (2.5%) | 30 (4.6%) | NA | NA |
| Disability |  |  |  |  |  |  |
| Yes, *N (%)* | NA | NA | 125 (6.6%) | 50 (8.3%) | NA | NA |
| No, *N (%)* | NA | NA | 1730 (90.7%) | 545 (87.4%) | NA | NA |
| Missing, *N (%)* | NA | NA | 50 (2.7%) | 25 (4.3%) | NA | NA |

*Note*. UKG – UK graduate; PMQ – Primary Medical Qualification; BAME - Black and Ethnic Minority.

**Supplementary Table 3.** Multivariable discrete-time survival (probability of completing training in six years) analysis results for the main cohort and the 2012-13 sub-cohort.

| **Predictor** *(reference category)* | **2012-17 cohort*** | | | **2012-13 sub-cohort*** | | |
| --- | --- | --- | --- | --- | --- | --- |
|  | **OR** | **95% CI** | ***p*-value** | **OR** | **95% CI** | ***p*-value** |
| Gender (*female*) | 0.671 | 0.593 - 0.759 | <0.001 | 0.743 | 0.621 - 0.889 | 0.001 |
| Region of PMQ (*non-UK*) | 0.463 | 0.408 - 0.525 | <0.001 | 0.471 | 0.391 - 0.566 | <0.001 |
| **Model statistics** | X^2^(7)=2390.004, *p* < 0.001  R^2^ = 0.307 (Cox & Snell)  R^2^ = 0.409 (Nagelkerke) | | | X^2^(7)=777.405, *p* < 0.001  R^2^ = 0.243 (Cox & Snell)  R^2^ = 0.324 (Nagelkerke) | | |

*Note*. *Dummy variables representing time variables were entered into the model as intercepts.

PMQ – Primary Medical Qualification.

**Supplementary Table 4** Hazards (probability of not progressing) and cumulative survival probabilities (probability of progressing to the next training level) for the main 2012-17 cohort and the 2012-13 sub-cohort according to gender and region of PMQ.

|  |  | **Hazards** | | | | **Cumulative survival** | | | |
| --- | --- | --- | --- | --- | --- | --- | --- | --- | --- |
|  | **Unstandardized coefficient** | **Non-UKG**  **ale** | **UKG**  **Male** | **UKG**  **Female** | **Non-UKG**  **Female** | **Non-UKG**  **Male** | **UKG**  **Male** | **UKG**  **Female** | **Non-UKG**  **Female** |
| **2012-17 cohort** |  |  |  |  |  |  |  |  |  |
| CT1-CT2 | -0.821 | 0.228 | 0.120 | 0.169 | 0.306 | 0.772 | 0.880 | 0.831 | 0.694 |
| CT2-CT3 | -0.802 | 0.231 | 0.122 | 0.172 | 0.310 | 0.594 | 0.773 | 0.688 | 0.479 |
| CT3-ST4 | 1.027 | 0.652 | 0.464 | 0.564 | 0.736 | 0.207 | 0.414 | 0.300 | 0.126 |
| ST4-ST5 | -0.812 | 0.229 | 0.121 | 0.170 | 0.307 | 0.159 | 0.364 | 0.249 | 0.088 |
| ST5-ST6 | -0.198 | 0.355 | 0.203 | 0.275 | 0.451 | 0.103 | 0.290 | 0.181 | 0.048 |
| **2012-13 sub-cohort** |  |  |  |  |  |  |  |  |  |
| CT1-CT2 | -0.75 | 0.260 | 0.142 | 0.182 | 0.321 | 0.740 | 0.858 | 0.818 | 0.679 |
| CT2-CT3 | -0.512 | 0.308 | 0.173 | 0.220 | 0.375 | 0.512 | 0.710 | 0.638 | 0.425 |
| CT3-ST4 | 0.976 | 0.664 | 0.481 | 0.555 | 0.726 | 0.172 | 0.368 | 0.284 | 0.116 |
| ST4-ST5 | -0.725 | 0.265 | 0.145 | 0.186 | 0.326 | 0.127 | 0.315 | 0.231 | 0.078 |
| ST5-ST6 | -0.251 | 0.366 | 0.214 | 0.268 | 0.438 | 0.080 | 0.247 | 0.169 | 0.044 |

*Note*. UKG – UK graduate.
